# 贝林妥欧单抗为基础的联合方案治疗CD19阳性系列模糊急性白血病的疗效分析

**DOI:** 10.3760/cma.j.cn121090-20240411-00133

**Published:** 2024-11

**Authors:** 小霞 吴, 贞 杨, 棽琪 陆, 馨慧 张, 爱宁 孙, 惠芬 周, 德沛 吴, 瞄 苗

**Affiliations:** 苏州大学附属第一医院、江苏省血液病研究所、国家血液系统疾病临床医学研究中心、国家卫生健康委员会血栓与止血重点实验室，苏州 215006 The First Affiliated Hospital of Soochow University, Jiangsu Institute of Hematology, National Clinical Research Center for Hematologic Diseases, Key Laboratory of Thrombosis and Hemostasis Under Ministry of Health, Suzhou 215006, China

## Abstract

系列模糊急性白血病（ALAL）是一种罕见的急性白血病类型，治疗难度极大。本文报道6例成功接受贝林妥欧单抗为基础的联合方案作为一线治疗的CD19阳性ALAL患者。其中5例为B/髓表型，1例为B/T表型。经过1个周期的贝林妥欧单抗联合治疗后，6例患者均获完全缓解（CR）。此外3例患者达到了流式细胞术微小残留病（MRD）阴性（<0.01％）；4例患者可评估分子学MRD，其中2例患者获得了分子学缓解。整个治疗中的最佳治疗反应：5例患者（83.3％）达到流式细胞术MRD阴性，4例有可评估分子学MRD的患者全部达到分子学缓解。中位随访时间为15个月，总生存率为100％，无事件生存率为83.3％。本研究初步证明贝林妥欧单抗为基础的联合方案是一线治疗CD19阳性ALAL患者的有效、安全疗法。

## Abstract (en)

Acute leukemia of ambiguous lineage（ALAL）is a rare type of acute leukemia and is extremely difficult to treat. Here, we present six patients with CD19-positive ALAL who were successfully treated with blinatumomab-based combination treatment in the front-line setting. Five were diagnosed with B-cell/myeloid mixed phenotype acute leukemia（MPAL）and one with B-cell/T cell MPAL. All six patients achieved complete remission after one cycle of blinatumomab combination treatment. Furthermore, 3（50％）patients achieved MRD-negative（<0.01％）by flow cytometry and 2（50％）of four patients with evaluable molecular MRD achieved molecular remission. At some point during the treatment, 5（83.3％）patients achieved MRD negativity and all four patients with evaluable molecular MRD had molecular remission. The overall survival was 100%, and the event-free survival was 83.3％ after a median follow-up time of 15 months. This study provides preliminary evidence that blinatumomab-based combination therapy is an effective and safe treatment for patients with CD19-positive ALAL in the front-line setting.

系列模糊急性白血病（ALAL）是一种罕见的急性白血病，约占所有急性白血病的3％[1]。据报道，与急性髓系白血病（AML）和急性淋巴细胞白血病（ALL）患者相比，ALAL患者的死亡风险分别增加了26％和59％[2]。有研究表明，与以AML为基础的疗法相比，以ALL为基础的疗法治疗新确诊的ALAL患者完全缓解（CR）率更高[3]。另一项研究显示，ALAL患者能从异基因造血干细胞移植（allo-HSCT）中获益[4]。然而，针对ALAL患者的有效、安全的治疗方案尚无定论。

贝林妥欧单抗是一种CD3/CD19双特异性单克隆抗体，已被美国食品药品管理局批准用于治疗复发/难治B-ALL[5]和微小残留病（MRD）阳性的B-ALL[6]。其安全性和有效性促使贝林妥欧单抗联合化疗在新诊断的Ph染色体阴性B-ALL患者中作为一线治疗应用[7]–[8]。研究表明，基于维奈克拉的联合用药治疗初治或复发/难治ALL和AML具有良好的协同治疗效果和可耐受的安全性[9]–[10]。贝林妥欧单抗与这些方案联合应用因具有独特和不同的作用机制，可能有助于ALAL的有效治疗。我们报告了在非临床试验环境下使用基于贝林妥欧单抗的联合方案成功治疗的6例CD19阳性ALAL患者。

## 病例与方法

1. 病例资料：回顾性分析2022年在我院以贝林妥欧单抗联合方案为一线治疗的6例CD19阳性ALAL患者的疗效及不良反应。6例患者均符合WHO（2022年版）或欧洲白血病网（2022年版）制定的ALAL诊断标准。所有患者均签署了知情同意书。

2. 方法：收集患者的年龄、性别、骨髓MICM分型、治疗方案、MRD、并发症、贝林妥单抗联合方案等资料。不良反应依据CTCAE 5.0标准，细胞因子释放综合征（CRS）分级依据Lee分级标准。骨髓完全缓解（CR）定义为骨髓原始幼稚细胞<5％，脱离输血，中性粒细胞绝对计数>1.0×10^9^/L，PLT>100×10^9^/L，无髓外白血病表现。流式细胞术MRD阴性定义为<1×10^−4^。

3. 统计学处理：采用描述性分析，计数资料采用例数（百分比）表示，非正态分布的计量资料采用中位数（范围）表示。

## 结果

1. 患者一般资料：6例患者的中位年龄为45.5（21～60）岁；男5例，女1例。B/髓ALAL 5例，其中4例为初诊，1例为接受了1个疗程的Hyper-CVAD诱导治疗后部分缓解的患者（骨髓原始细胞占10％）；1例患者为初诊的B/T表型ALAL。2例患者携带KMT2A重排，其中1例患者同时携带FLT3-ITD突变。1例患者伴ETV6-ABL1和KMT2A部分串联重复（PTD），1例患者伴BCR-ABL1和IKZF1缺失（表1）。

**表1:** 6例CD19阳性系列模糊急性白血病患者的临床特征

例号	年龄（岁）	性别	流式细胞术免疫表型	诊断	WBC（×10^9^/L）	骨髓原始细胞（％）	染色体核型	分子学异常
1	51	男	HLA-DR、CD19、CD33、CD117、CD11b、CD64、CD79a	B/髓	10.5	67	46,XY,t（9；11）（p22；q23）add（2）（p25）	KMT2A-MLLT3
2	60	女	CD34、CD19、CD13、CD33、CD117、MPO、CD79a、CD25	B/髓	138.8	87	46,XX,t（10；11）（q12；q23）	KMT2A-TET1, FLT3-ITD
3	43	男	CD7、CD34、HLA-DR、CD19、CD13、CD33、CD117、CD79a	B/髓	1.8	52	46,XY,del（20）（q12q13）	KMT2A-PTD, ETV6-ABL1
4	29	男	CD34、HLA-DR、CD10、CD19、CD13、CD33、CD15、CD38、cCD79a、CD22	B/髓	1.2	10	正常核型	阴性
5	21	男	CD34、HLA-DR、CD10、CD19、CD13、CD33、CD38、cMPO、cCD79a、CD25	B/髓	102.0	76	未做	BCR-ABL1, IKZF1缺失
6	48	男	CD7、CD19、CD2、CD117、CD38、CD8、CD79a、cCD3	B/T	4.8	40	正常核型	阴性

2. 治疗方案：所有患者均接受了7 d的糖皮质激素作为前期治疗（其中2例还接受了白细胞清除术和羟基脲降白细胞）。在诱导治疗期间，4例患者接受了贝林妥欧单抗联合维奈克拉±阿扎胞苷治疗，1例Hyper-CVAD诱导治疗后部分缓解的患者接受了贝林妥欧单抗联合阿扎胞苷治疗，1例BCR-ABL1阳性患者接受了贝林妥欧单抗联合奥雷巴替尼治疗（表2）。贝林妥欧单抗的剂量为9 µg/d，持续1周，然后28 µg/d，持续3周；因与伏立康唑联用，维奈克拉的剂量为100 mg/d，持续14～21 d；阿扎胞苷的皮下注射剂量为 75 mg·m^−2^·d^−1^，持续7 d；奥雷巴替尼的剂量为40 mg，隔日1次。为预防中枢神经系统浸润，每4～6周接受1次阿糖胞苷（40 mg）、甲氨蝶呤（15 mg）及地塞米松（5 mg）的鞘内注射治疗。

3. 疗效：经过1个周期的贝林妥欧单抗联合治疗，6例患者均达到CR，3例患者达到流式细胞术MRD阴性；4例患者可评估分子学MRD，其中2例患者达到分子学缓解（表2）。接受1个周期化疗方案为主的巩固治疗后，2例MRD持续阳性的患者［1例流式细胞术MRD水平0.026％，另1例分子学MRD（BCR-ABL1）水平为0.25％］，在接受第2个周期的贝林妥欧单抗联合治疗后，MRD均达到阴性。

**表2:** 6例CD19阳性系列模糊急性白血病患者治疗经过及微小残留病（MRD）

例号	诱导治疗		第1周期巩固治疗		第2周期巩固治疗		后期巩固治疗	移植前状态	移植方式
	方案	MRD	方案	MRD	方案	MRD			
1	BiTE+VEN	CD19^+^:0.012％	Hyper-CVAD+AZA	CD19^+^: 0.026％	BiTE+VEN	CD19^+^: 0％	MTX	CD19^+^: 0％	HRD
		MLL-AF9: 0.0％		MLL-AF9: 0.0％		MLL-AF9: 0.0％		MLL-AF9: 0.0％	
2	BiTE+VEN+AZA	CD19^+^: 0％	Hyper-CVAD+VEN	CD19^+^: 0％	复发^a^	CD19^dim^ AML	AZA+VEN+吉瑞替尼	MLFS	HRD
		FLT3-ITD: 0.0％		FLT3-ITD: 0.0％		FLT3-ITD（+）		FLT3-ITD^b^	
		MLL-TET1: 0.0％		MLL-TET1: 0.0％		MLL-TET1（+）		MLL-TET1^b^	
3	BiTE+VEN	CD19^-^:0.14％	Hyper-CVAD +VEN	CD19^-^: 0.015％	MA+VEN	CD19^-^:0.015％	Hyper-CVAD+VEN	CD19^-^: 0.024％	HRD
		MLL-PTD: 0.41％		MLL-PTD: 0.07％		MLL-PTD: 0.0％	AZA+VEN	MLL-PTD: 0.0％	
		ETV6-ABL1: 0.0％		ETV6-ABL1: 0.0％		ETV6-ABL1: 0.0％	MTX	ETV6-ABL1: 0.0％	
4	BiTE+AZA	CD19^-^CD22^+^: 0.03％	AZA+INO	CD19^+^CD22^-^: 0％	ID-Ara-C	CD19^+^CD22^-^: 0％	BiTE	CD19^+^CD22^-^: 0％	HRD
5	BiTE+奥雷巴替尼	CD19^+^: 0％	Hyper-CVAD +奥雷巴替尼	CD19^+^: 0％	BiTE +奥雷巴替尼	CD19^+^: 0％	MA+奥雷巴替尼	CD19^+^: 0％	/
		BCR-ABL1: 0.88％		BCR-ABL1:0.25％		BCR-ABL1: 0.0％	HD-MTX+伊马替尼	BCR-ABL1: 0.0％	
							Hyper-CVAD+伊马替尼		
6	BiTE+VEN+AZA	CD19^+^: 0％	CLAG	CD19^-^:0.71％	AZA+VEN	CD19^+^: 0％	/	CD19^+^: 0％	MUD

**注** ^a^因治疗延迟>2个月复发，并转为CD19弱表达的髓系表型；^b^细胞数太少无法分析。BiTE：贝林妥欧单抗；VEN：维奈克拉；AZA：阿扎胞苷；Hyper-CVAD：大剂量环磷酰胺+表阿霉素+长春地辛+地塞米松；INO：奥加伊妥珠单抗；MA：中剂量甲氨蝶呤+阿糖胞苷；HD-MTX：大剂量甲氨蝶呤；ID-Ara-C：中剂量阿糖胞苷；HRD：半相合移植；MUD：无关全相合移植；/：未进行

2例患者在接受1个周期的贝林妥欧单抗联合治疗后表现为CD19阴性的MRD。例3的ETV6-ABL1和KMT2A-PTD检测结果为阴性，在接受多药化疗多个周期后，流式细胞术MRD仍为阳性（<0.1％）；例4为CD19阴性、CD22阳性的MRD（0.03％），在接受调整剂量的奥加伊妥珠单抗（1 mg/周）联合阿扎胞苷治疗2周后，MRD达到阴性。整个治疗中的最佳治疗反应：5例（83.3％）患者达到了流式细胞术MRD阴性，而4例可评估分子学MRD的患者全部达到了分子学缓解。

例2因个人原因延迟治疗达2个多月而复发，其余患者持续缓解。例2同时伴有MLL-TET1和FLT3-ITD突变，复发后表现出向髓系的转变（CD13、CD33、CD34、MPO、CD38和CD64阳性，CD19弱表达），接受了1个周期的维奈克拉和吉瑞替尼治疗后，该患者达到了形态学无白血病状态，随后接受了allo-HSCT。

除BCR-ABL1阳性的患者外，其余5例（83.3％）患者均接受了allo-HSCT，其中4例为单倍体造血干细胞移植，另1例为无关供者造血干细胞移植。截至2023年10月31日，中位随访15个月，6例患者的总生存（OS）率为100％，无事件生存（EFS）率为83.3％。

4. 安全性分析：在诱导治疗期间，最常见的3级或4级不良事件为血小板减少（4例）、中性粒细胞减少（6例）和贫血（6例）。1例患者在治疗前有3级肛周感染，在治疗过程中逐渐恢复；未观察到其他严重感染情况。2例患者有1级CRS，1例患者有1级头痛。没有严重的CRS、神经毒性或导致治疗中断的不良事件发生。1例患者治疗前有低纤维蛋白原血症，考虑与本病相关，在前期激素治疗期间进展为弥散性血管内凝血（DIC），诱导治疗过程中接受纤维蛋白原和成分血输注支持，贝林妥欧单抗联合治疗1周后DIC得到改善，未发生任何大出血事件。

## 讨论

在本研究中，我们报道了使用贝林妥欧单抗联合疗法成功治疗6例CD19阳性ALAL患者的情况。在之前的报道中，贝林妥欧单抗被用作MRD阳性ALAL患者移植前的桥梁[11]–[12]。同样有研究表明，贝林妥欧单抗治疗或与供者淋巴细胞输注联合应用对移植后复发的ALAL患者同样显示出了有效性和安全性[13]–[14]。另一项关于2例初治ALAL患者的研究表明，维奈克拉与去甲基化药物（地西他滨或阿扎胞苷）联合是一种潜在的治疗选择[15]。此外，Bacchiarri等[16]报道1例多发性骨髓瘤患者继发了难治性ALAL，在接受了贝林妥欧单抗、维奈克拉联合阿扎胞苷的治疗后，达到了CR。

在我们的研究中，除1例BCR-ABL1阳性患者和1例经1个疗程Hyper-CVAD方案诱导治疗后部分缓解的患者，其余4例患者均接受了贝林妥欧单抗联合维奈克拉±阿扎胞苷的一线治疗，4例患者均在诱导治疗后达到了CR，没有出现严重的CRS、神经毒性或导致治疗中断的不良事件。此为目前报道的首项贝林妥欧单抗与维奈克拉±阿扎胞苷联合用于一线治疗CD19阳性ALAL的研究，为临床医师治疗ALAL提供了参考。

2016年WHO把ALAL细分为两个基因组类别：BCR-ABL1融合基因阳性和KMT2A重排。在更新后的2022年分类中，新增了ZNF384和BCL11B重排两种ALAL亚型。准确的基因组分析可以为诊断、风险分级和治疗决策提供最佳指导。在本研究中，有4例患者的细胞遗传学为中危或高危，尽管如此，所有6例患者在诱导治疗结束时均达到了CR。对于BCR-ABL1阳性的ALAL患者，强烈建议添加酪氨酸激酶抑制剂（TKI）[17]。本研究中的BCR-ABL1阳性ALAL患者接受了贝林妥欧单抗与奥雷巴替尼联合治疗，随后化疗联合TKI与贝林妥欧单抗联合TKI交替巩固治疗，在第二轮贝林妥欧单抗治疗后获得了分子学缓解，迄今在未行造血干细胞移植情况下已持续缓解10个月。KMT2A重排的ALAL患者可在淋系和髓系表型之间转换，这是KMT2A重排ALAL患者侵袭性强、预后差的重要原因之一[18]。本研究中2例携带KMT2A重排的患者分别在第一轮和第二轮贝林妥欧单抗治疗后实现了MRD阴性和分子学缓解，遗憾的是，其中1例患者因治疗延误而复发，并转换为髓系表达。

一项涉及233例ALAL儿童患者的回顾性多国研究报告显示[19]，诱导治疗后原始细胞≥5％的患者的5年EFS率较差（<50％），在采用ALL方案治疗12周结束时CD19阳性且MRD阳性的患者的EFS率为（50±19）％（>0.01％），而整个CD19阳性患者的EFS率为（83±5.3）％，表明早期清除MRD预示着更好的生存。在我们的研究中，诱导治疗后6例（100％）患者达到了CR，3例（50％）达到了流式细胞术MRD阴性，4例可评估分子学MRD的患者中有2例（50％）达到了分子学缓解。2例患者在接受第二个周期的贝林妥欧单抗治疗后达到了MRD阴性。总体而言，在第2周期巩固治疗后，6例患者中有5例（83.3％）在某个时间点达到了流式细胞术MRD阴性，所有4例可评估分子MRD的患者均实现了深度分子学缓解。

一些患者在贝林妥欧单抗治疗1个周期后MRD阴性，在第2个周期后会出现MRD转为阳性，或出现CD19阴性的复发。在本研究中，随后的巩固治疗改为序贯化疗和贝林妥欧单抗（如果CD19流式细胞术MRD阳性或分子学MRD持续阳性）。除例2因延误治疗复发外，其他患者均未复发。诱导后CD19阴性、CD22阳性MRD的例4接受了奥加伊妥珠单抗联合阿扎胞苷治疗，并获得了MRD缓解。表明序贯其他治疗方案可能有利于预防贝林妥欧单抗治疗后的复发。

本研究结果初步显示基于贝林妥欧单抗的无化疗诱导联合疗法一线治疗CD19阳性ALAL患者安全、有效。本研究样本量较小，且诱导方案缺乏统一性；另一方面，由于ALAL这个疾病类型的高危性，除BCR-ABL1阳性的患者外，其余患者均接受了allo-HSCT，缺乏后期贝林妥欧单抗长期治疗的临床数据。故最优联合用药方案、贝林妥欧单抗后期维持治疗的有效性等尚需前瞻性、多中心的临床研究来进一步探索。
